# Impact of Cardiovascular Risk Factors on the Occurrence of Cardiovascular Events in Antineutrophil Cytoplasmic Antibody (ANCA)-Associated Vasculitides

**DOI:** 10.3390/jcm10112299

**Published:** 2021-05-25

**Authors:** Camille Roubille, Soledad Henriquez, Cédric Mercuzot, Claire Duflos, Bertrand Dunogue, Karine Briot, Loic Guillevin, Benjamin Terrier, Pierre Fesler

**Affiliations:** 1 Department of Internal Medicine, Lapeyronie Hospital, Montpellier University Hospital, 34000 Montpellier, France; c-mercuzot@chu-montpellier.fr (C.M.); p-fesler@chu-montpellier.fr (P.F.); 2 PhyMedExp, University of Montpellier, INSERM U1046, CNRS UMR 9214, 34000 Montpellier, France; 3 Department of Internal Medicine, Assistance Publique Hôpitaux de Paris-Centre, Université de Paris, Hôpital Cochin, 75014 Paris, France; soledad.henriquez@aphp.fr (S.H.); bertrand.dunogue@aphp.fr (B.D.); loic.guillevin@aphp.fr (L.G.); benjamin.terrier@aphp.fr (B.T.); 4 Université de Paris, 75006 Paris, France; karine.briot@aphp.fr; 5 Clinical Research and Epidemiology Unit, Medical Information Department, Lapeyronie Hospital, Montpellier University Hospital, 34000 Montpellier, France; c-duflos@chu-montpellier.fr; 6 Department of Rheumatology, Assistance Publique Hôpitaux de Paris-Centre, Université de Paris, Hôpital Cochin, 75014 Paris, France

**Keywords:** cardiovascular risk factors, antineutrophil cytoplasmic antibody (ANCA)-associated vasculitis, cardiovascular risk, hypertension, dyslipidemia, sedentary lifestyle

## Abstract

Despite improvement in the prognosis of ANCA-associated vasculitides (AAVs), increased mortality, mainly from a cardiovascular origin, persists. We aimed to determine the role of cardiovascular risk factors (CVRFs) on the occurrence of major cardiovascular events (MACEs) in AAVs. Patients with AAVs were successively included in a prospective cohort study, which assessed CVRFs (defined by age >50 years in men and >60 years in women, personal history of cardiovascular disease, smoking status, obesity, diabetes, dyslipidemia, hypertension, and sedentary lifestyle), the use of glucocorticoids and immunosuppressive agents at baseline and during follow-up, and the occurrence of MACEs. One hundred and three patients were included, with a median follow-up time of 3.5 years. In the glucocorticoids and cyclophosphamide adjusted multivariate analysis, the occurrence of MACEs was associated with older age (*p* = 0.001, OR = 14.71, 95% CI (confidence interval) = 2.98–72.68), cardiovascular history (*p* = 0.007, OR (odds ratio) = 6.54, 95% CI = 1.66–25.71), sedentary lifestyle (*p* = 0.011, OR = 4.50, 95% CI = 1.42–14.29), hypertension (*p* = 0.017, OR = 5.04, 95% CI = 1.33–19.12), and dyslipidemia (*p* = 0.03, OR = 3.86, 95% CI = 1.14–13.09). The occurrence of MACEs was associated with the number of CVRFs (*p* < 0.001), but not with the use of glucocorticoids or cyclophosphamide (*p* = 0.733 and *p* = 0.339, respectively). The implementation of a screening and management program for modifiable CVRFs, particularly hypertension, sedentary lifestyle, and dyslipidemia, may be beneficial for AAV patients in order to reduce their cardiovascular risk.

## Significance and Innovations

- Despite improvement in the prognosis of ANCA-associated vasculitides (AAVs), increased mortality, mainly from a cardiovascular origin, persists. This 3-year cohort study revealed that the occurrence of MACEs (major cardiovascular events) in AAVs was associated with older age, the presence of a history of CVD (cardiovascular diseases), dyslipidemia, hypertension, and a sedentary lifestyle.
- The implementation of a screening and management program for modifiable CVRFs (cardiovascular risk factors), particularly hypertension, a sedentary lifestyle, and dyslipidemia, may be beneficial for AAV patients in order to reduce their cardiovascular risk.
- While a tight control of AAV inflammation is required to prevent CVD, traditional CVRFs should not be overlooked. The specific management of cardiovascular risk should combine the control of AAV disease activity and traditional CVRFs.

## 1. Introduction

Antineutrophil cytoplasmic antibody (ANCA)-associated vasculitides (AAVs) are a heterogeneous group of inflammatory diseases that includes granulomatosis with polyangiitis (GPA, Wegener’s granulomatosis), microscopic polyangiitis (MPA), and eosinophilic granulomatosis with polyangiitis (EGPA, Churg-Strauss syndrome), and is characterized by small vessel necrotizing vasculitis and the presence of autoantibodies targeting either proteinase 3 (PR3) or myeloperoxidase (MPO). It can damage different organs, especially the kidneys; the lungs; the central or peripheral nervous system; the ear, nose, and throat; the skin; the heart.

Thanks to the current immunosuppressive (IS) treatments, AAV’s overall mortality [1] has decreased over the last few decades [2,3,4,5], but remains 2.7 times higher than in the general population [3]. This excess mortality is not only related to the disease itself but is also induced by treatment complications [6] and comorbidities [7]. The presence of comorbidities at diagnosis of AAV increases the risk of death at 2 years, justifying their screening and management [7]. In particular, cardiovascular diseases (CVDs) [4,8,9] are the leading cause of death after the first year following diagnosis [10,11]. Major cardiovascular events (MACEs) occur 1.65 to 3.15 times more frequently in AAV patients than in the general population [12], with an increased risk of coronary artery disease [13,14,15,16,17] and stroke [13,18], where the latter occurs 1.08 to 8.49 times more frequently than in the general population [12].

The pathophysiology of this increased cardiovascular risk can be related to various mechanisms. For example, ANCAs may trigger the release of bioactive molecules that have a role in thrombosis, inflammation, and endothelial activation [19]. This endothelial dysfunction is more prevalent in AAV patients as compared to the general population [20,21,22], is associated with the presence of inflammation proteins, particularly during vasculitis flares [21,22], and contributes to fibrosis, thrombosis, and atherosclerosis, beginning with preclinical target organ damage followed by overt CVD [23,24]. In EGPA patients, eosinophils could also have a direct role in endothelial cell injury [22]. Furthermore, therapeutics may have some specific impact, especially glucocorticoids (GCs), which are often considered by physicians as partially responsible for such an increased cardiovascular risk [25]. However, traditional cardiovascular risk factors (CVRFs) should not be ignored [26,27], although their specific role has not yet been demonstrated.

The aim of our study was to assess the impact of CVRFs on the occurrence of MACEs in AAVs by investigating the relationship between CVRFs and the occurrence of MACEs at 3 years in the prospective OSTEOVAS cohort.

## 2. Methods

### 2.1. Study Population and Setting

In the OSTEOVAS cohort, a monocentric prospective observational cohort (Cochin Hospital, Paris, France), patients with an AAV were consecutively included between January 2014 and May 2015 in a longitudinal routine care study assessing cardiovascular complications and other sequelae and were prospectively followed up on, as described before [28]. Patients fulfilled the American College of Rheumatology (ACR) criteria for GPA and EGPA [29,30], and/or the European Medicines Agency (EMA) algorithm and/or Chapel Hill definitions for all forms of vasculitis, including MPA [31]. Informed consent was obtained from all patients. Patients suffering from polyarteritis nodosa (PAN) were not included in the present study. The protocol of this study was approved by the Ethical Review Committee of the Cochin University Hospital (CLEP) (No. AAA-2021-08025).

### 2.2. Data Collection at Baseline

Some clinical and functional data were recorded: physical examination, including weight and height, medical history, age, gender, and disease duration. Treatments were listed, including GCs (current dose and cumulative dose of prednisone equivalent), IS or immunomodulatory agents, and other therapies, such as aspirin, statins, anti-diabetics, and anti-hypertensive therapies. The Birmingham Vasculitis Activity Score (BVAS) version 3 [32] and the Vasculitis Damage Score (VDI) score [33] were used to evaluate the activity and the damage related to the AAVs at baseline. Disability and health-related quality of life were respectively assessed using the health assessment questionnaire (HAQ) [34] and the 36-item Short-Form Health Survey (SF-36) [35]. Biological parameters, including ANCA status and specificity (stratified as PR3-ANCA or MPO-ANCA), glucose and lipids levels, glycated hemoglobin, and C-reactive protein (CRP), were assessed at the inclusion.

CVRFs were identified, including older age depending on gender (>50 years for men or >60 years for women), personal medical history of CVD, smoking status (non-smoker or former/current smoker), obesity (body mass index (BMI) ≥ 30 kg/m^2^), diabetes mellitus (defined as fasting blood glucose ≥ 7 mmol/L or use of anti-diabetic drugs), dyslipidemia (defined as total cholesterol ≥ 5.18 mmol/L, LDL cholesterol ≥ 3.30 mmol/L, or use of lipid-lowering drugs), hypertension, and sedentary lifestyle. Comorbidities were declarative data, except for hypertension (presence when blood pressure measured ≥ 140/90 mmHg or when participants received anti-hypertensive drugs). They were defined as “present” or “absent” at inclusion. The sum of the number of CVRFs that were present was calculated for each patient.

### 2.3. Outcomes and Follow-Up

A 3-year prospective follow-up was carried out, during which the occurrence of MACEs was noted, defined as stroke or transient ischemic attack (TIA), myocardial infarction, unstable angina, arterial revascularization, and/or hospitalization for or death from cardiovascular causes. Current treatment at the time of follow-up (GCs and/or IS treatment) was noted. GCs or cyclophosphamide use was considered if the patient was receiving GCs or cyclophosphamide either at baseline or at follow-up. These two treatments were included as adjusting factors in the multivariate analysis given their potential cardiovascular toxicity [25,36].

### 2.4. Statistics

The quantitative variables are expressed as mean +/− standard deviation or median and interquartile range (IQR) in the case of a skewed distribution. Qualitative variables are expressed as a frequency. We performed a binary logistic regression to assess the association between each CVRF, BVAS, and CRP (both parameters expressed as above or below the median) and the occurrence of MACEs, adjusting for the presence or absence of GCs and cyclophosphamide either at baseline or during follow-up. We also evaluated the association between the number of CVRFs present and the MACEs’ occurrences, still adjusting for the presence or absence of GCs and cyclophosphamide. The results of the logistic regression are expressed as an odds ratio (OR) and confidence intervals (95% CI). The statistics were performed using IBM SPSS Statistics 25 software (IBM, Armonk, NY, USA). A *p*-value < 0.05 was considered to be statistically significant.

## 3. Results

### 3.1. Study Population

The study population comprised 103 patients, including 57 women, mean age of 52.9 ± 17.4 years (Table 1). The median duration of vasculitis was 4.5 years (IQR (interquartile range) 0.9; 8.3). Twelve patients (11.6%) had MPA, 62 (60.2%) had GPA, and 29 (28.1%) had EGPA. Twenty-five (24.3%) had MPO-ANCA and 44 (42.7%) had PR3-ANCA.

At baseline, 17 (16.5%) were obese, 54 (52.4%) had hypertension, 7 (6.8%) had diabetes mellitus, 19 (18.5%) had dyslipidemia, 11 (10.7%) had a past history of CVD, 43 (41.8%) were current or past smokers, and 20 (19.4%) had sedentary lifestyles. Forty-six patients (44.7%) were men over 50 years of age or women over 60 years of age. Among the 54 patients with hypertension, 16 (29.6%) did not receive any antihypertension treatment.

Eighty-six patients (83.50%) were taking GCs, and 77 (74.76%) were on IS agents, of whom 13 (12.62%) were taking cyclophosphamide.

### 3.2. Outcomes

The median follow-up was 3.5 years (IQR 2.9; 3.9). Sixteen patients (15.5%) had at least one MACE during follow-up, including three strokes or TIA, three coronary revascularizations, nine hospitalizations for cardiovascular causes, and one cardiovascular death. One patient had two MACEs, and one had three, while all the others had only one. Only three patients were lost to follow-up. There were six deaths, one of which was of cardiovascular origin. The distribution of MACEs according to the number of CVRFs is presented in Table 2.

**Figure 1:**
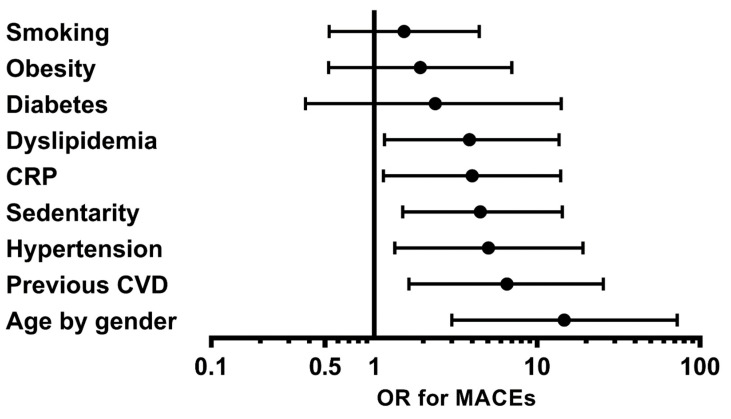
Odds ratios of presenting a major cardiovascular event during follow-up for each cardiovascular risk factor and CRP from the OSTEOVAS cohort. The multivariate analysis was adjusted for the use of glucocorticoids and cyclophosphamide. CRP, C-reactive protein; CVD, cardiovascular disease; OR, odds ratio; MACEs, major cardiovascular events; age by gender: being >50 years for men and >60 years for women.

Sixty-four patients (62.1%) were still on GCs at follow-up, and 51 (49.51%) were on IS treatment, with only one patient on cyclophosphamide. In the multivariate analysis, older age was significantly associated with the occurrence of MACEs (*p* = 0.001, OR = 14.71, 95% CI = 2.98–72.68), as well as a personal medical history of CVD (*p* = 0.007, OR = 6.54, 95% CI = 1.66–25.71), a sedentary lifestyle (*p* = 0.011, OR = 4.50, 95% CI = 1.42–14.29), hypertension (*p* = 0.017, OR = 5.04, 95% CI = 1.33–19.12), and dyslipidemia (*p* = 0.03, OR = 3.86, 95% CI = 1.14–13.09) (Table 3) (Figure 1), while the other CVRFs (smoking status, obesity, diabetes mellitus) were not associated with the occurrence of MACEs (*p* = 0.44, *p* = 0.321, and *p* = 0.34, respectively).

Interestingly, CRP level > median was significantly associated with the occurrence of MACEs (*p* = 0.028, OR = 4.028, 95% CI = 1.16–13.98) (Figure 1), whereas BVAS was not (*p* = 0.319). Moreover, the occurrence of MACEs was significantly associated with the number of CVRFs present (*p* < 0.001, OR 1.74, 95% CI = 1.28–2.37), but not with the presence of GC (*p* = 0.733) or cyclophosphamide (*p* = 0.339) in the multivariate analysis (Table 4).

## 4. Discussion

Our study explored the impact of each CVRF on the occurrence of MACEs, finding a major role of age since an age above 50 years for men and above 60 years for women increased the risk of a MACE occurrence by almost 15 times. Similarly, Haris et al. had shown that in elderly people (over 65 years of age in his study), the increased mortality observed in AAVs was mostly from a cardiovascular origin [37]. The presence of a personal history of CVD also had a 6.5-fold increase in cardiovascular risk. A retrospective cohort study identified these two CVRFs as predictive factors for the occurrence of arterial thrombotic events in a multivariate analysis [27].

Importantly, some modifiable CVRFs, including sedentary lifestyle, hypertension, and dyslipidemia, were also associated with the occurrence of MACEs, with ORs of 4.5, 5, and 3.9, respectively. AAV patients present a higher prevalence of hypertension up to 2.45 times than in the general population [12,38,39] and experience changes in their lipid profile [40]. While we found an association between dyslipidemia and the occurrence of MACEs, we did not find an association with obesity. Although BMI is recommended to identify a patient at higher cardiovascular risk, it has some limitations [41]. Indeed, it has been reported that nearly one-third of obese adults do not have cardiometabolic diseases [42]. This argues for the need to go beyond a simple BMI assessment, which involves including other surrogate markers for CV risk. For instance, higher visceral adipose tissue (VAT) mass has been associated with an increased risk of developing CVD, independently of BMI [43]. Abdominal adipose tissue measurement may be predictors of incident MACEs in AAV patients [28]). Moreover, although diabetes was not associated with the occurrence of MACEs in the present study, likely due to a lack of power (only seven patients were diabetic in the cohort), its prevalence is increased in AAVs [38,39], ranging from 7.2 to 29% [12], and also justifies its detection and management. Therefore, the role of these CVRFs and their increased prevalence justifies their imperative screening.

Interestingly, the results of the multivariate analysis showed that the number of CVRFs was associated with the occurrence of MACEs. This result highlights the primordial role of screening and management of these CVRFs in order to reduce their deleterious impact and has direct implication in the daily management of AAV patients. Indeed, given that among the CVRFs we found to be associated with the occurrence of MACEs, hypertension, dyslipidemia, and a sedentary lifestyle are modifiable risk factors; as such, clinicians can act and decrease the weight of each CVRF and thus reduce the overall cardiovascular risk. The screening and management of these CVRFs should therefore be considered a major issue, in addition to vasculitis control.

In contrast to preconceived ideas, the use of GCs or cyclophosphamide did not increase the risk of MACEs in our study. GCs are widely used in AAV management and their benefit/risk ratio is still controversial, particularly its cardiovascular impact, like in other inflammatory diseases. For instance, in rheumatoid arthritis, one meta-analysis reported that GCs usage was associated with an increased risk of all cardiovascular events (relative risk = 1.47, 95% CI = 1.34–1.60), including myocardial infarction, heart failure, and stroke [44]. The absence of a link between GCs and the occurrence of MACEs in our study could be related to the better control of the inflammatory disease thanks to GCs [45], leading to a decrease in systemic inflammation and the subsequent inflammation-related atherosclerosis, counterbalancing the pro-atherogenic effects of GCs.

In addition, we found a significant association between the occurrence of MACEs and the number of CVRFs. While arguing for a relevant impact of CVRFs on AAV cardiovascular risk, we can hypothesize that CVRFs alone do not entirely account for the increased cardiovascular risk. Indeed, compared to other studies conducted in the general population, such as the Interheart study exploring the effect of CVRFs on coronary heart disease risk in which CVRFs accounted for most of the risk [46], here the relative effect of the number of CVRFs appeared to be smaller, although significant. Thus, the effect of systemic inflammation due to the vasculitis itself may have a synergic impact on cardiovascular risk in addition to CVRFs, leading to premature atherosclerosis, as suggested by our results with a significant association between MACE occurrences and higher CRP levels. Indeed, inflammation is the cornerstone of both atherosclerosis and AAV. Atherosclerosis is no longer considered a condition in which lipids are being deposited passively in the arterial wall but rather fully recognized as an inflammatory complex disease [47]. Several studies have shown that subclinical atherosclerosis, as assessed using the carotid intima-media thickness (IMT), was greater in AAV compared to control patients [23].

Thanks to our results, the implementation of a systematic screening and management program for CVRFs seems to be necessary in order to improve the overall management and, ultimately, the survival of AAV patients. The overall management can be significantly improved. The study from Bramlage et al. showed that among the AAV patients with an indication for statin (72.5% of patients included), only 24.3% received it. Similarly, the blood pressure target of 130/80 mmHg (in the presence of proteinuria or chronic renal failure) was unmet in 65% of patients [48]. Current recommendations should be followed, ensuring that target blood pressure ranges are met while avoiding excessive blood pressure lowering in these potentially fragile patients [49,50]. Similar results were found in Houben’s study, with 36% of patients failing to reach their blood pressure target and 25% not receiving the indicated lipid-lowering treatment [51]. Therefore, screening and rigorous management of CVRFs can clearly be enhanced, particularly for hypertension and dyslipidemia, but also for a sedentary lifestyle. Furthermore, a randomized clinical trial is currently underway that aims to evaluate the benefit of an intervention program of physical activity techniques compared to no intervention on fatigue in patients with AAV [52]. This type of program could therefore also be beneficial in reducing sedentary lifestyles and improve the overall cardiovascular prognosis. In comparison, patients with rheumatoid arthritis are considered to be at high cardiovascular risk, with a 48% [53] increase in the prevalence of CVD and a 60% [54] increase in cardiovascular mortality compared to the general population. A clinical trial showed a decrease in the progression of subclinical atherosclerosis, as estimated using IMT, thanks to an intensive CVRF management program (treat-to-target) compared to conventional CVRF management during follow-up in patients with rheumatoid arthritis [55]. This type of intervention should be evaluated regarding AAV to improve the overall prognosis.

The limitations of our study include potential confounding factors that could not be taken into account. The adjustment of our analyses was based on the fact that cyclophosphamide and GCs were taken either at diagnosis or during follow-up and not on the cumulative dose of GCs due to the unavailability of these data during follow-up. Nevertheless, the continuation of GCs or cyclophosphamide may reflect vasculitis activity, thus providing an interesting adjustment parameter to consider. Moreover, the sample size of diabetes patients was low, leading to a lack of power that did not allow for the threshold of significance to be reached. Our study had some strengths. First, it was a prospective cohort study with a large number of patients with these rare diseases. There were very few lost to follow-up (only three patients). Despite its non-interventional nature, it is to our knowledge the first study to highlight the direct impact of CVRFs on the occurrence of MACEs in AAV patients.

In summary, this 3-year cohort study revealed that the occurrence of MACEs in AAV was associated with older age, the presence of a history of CVD, dyslipidemia, hypertension, and a sedentary lifestyle. The occurrence of MACEs was also associated with the number of CVRFs and with higher CRP levels. While a tight control of AAV inflammation is required to prevent CVD, traditional CVRFs should not be overlooked. The specific management of cardiovascular risk should combine the control of AAV disease activity and traditional CVRFs. The implementation and evaluation of a management program for modifiable CVRFs, including hypertension, dyslipidemia, and sedentary lifestyle, may therefore be beneficial for AAV patients.

## Figures and Tables

**Table 1:** Baseline demographic, clinical, biological, and functional characteristics of the study population from the OSTEOVAS cohort.

	*N*	Total Study Population (*n* = 103)
*Demographics and clinical parameters*		
Age (years) (mean SD)	103	52.88 ± 17.40
Male, *n* (%)	103	46 (44.66)
*Cardiovascular risk factors*		
Older age (>50 years for men, >60 years for women), *n* (%)	103	46 (44.66)
BMI (kg/m^2^) (mean SD)	103	25.34 ± 4.87
BMI > 30 kg/m^2^, *n* (%)	103	17 (16.5)
Diabetes mellitus, *n* (%)	103	7 (6.8)
Hypertension, *n* (%)	103	54 (52.4)
Ever smokers, *n* (%)	103	43 (41.8)
History of CVD, *n* (%)	103	11 (10.7)
Dyslipidemia, *n* (%)	103	19 (18.5)
Sedentary lifestyle (yes), *n* (%)	103	20 (19.4)
*Comorbidities and health-related scores*		
Osteoporosis, *n* (%)	103	28 (27.2)
SF-36 score (mean SD)	53	
Physical score		41.69 ± 10.21
Mental score		42.10 ± 9.86
*Vasculitis characteristics*		
Disease duration (months) (median, IQR)	103	54.05 (11.11; 99.14)
MPO-ANCA, *n* (%)	103	25 (24.3)
PR3-ANCA, *n* (%)	103	44 (42.7)
BVAS score (mean SD)	103	4.50 ± 8.31
VDI score (mean SD)	103	2.30 ± 2.05
HAQ score (mean SD)	60	0.31 ± 0.45
*Treatments*		
Use of GC, *n* (%)	103	86 (83.50)
Daily current dose of GC, (mg) (median IQR)	86	12 (5; 30)
Cumulative dose of GC, (g) (median IQR)	100	11.26 (6.00; 21.42)
Current immunosuppressive agents, *n* (%)	103	77 (74.76%)
Aspirin, *n* (%)	103	17 (16.5)
Statins, *n* (%)	103	16 (15.53)
Anti-hypertensive agents, *n* (%)	103	39 (37.86)
Anti-diabetics, *n* (%)	103	7 (6.80)
*Biological characteristics*		
CRP (mg/L) (mean SD)	99	9.15 ± 18.59
Hb1Ac (mean SD)	92	5.66 ± 0.75
LDL cholesterol (mean SD)	94	1.19 ± 0.45
HDL cholesterol (mean SD)	96	0.74 ± 0.29
Ratio proteinuria/creatininuria (mean SD)	96	29.98 ± 65.99

SD, standard deviation; IQR, interquartile range; ANCA, antineutrophil cytoplasmic antibody; BMI, body mass index; BVAS, Birmingham Vasculitis Activity Score; CRP, C-reactive protein; CVD, cardiovascular diseases; GC, glucocorticoids; HAQ, Health Assessment Questionnaire; MPO, myeloperoxidase; PR3, proteinase 3; VDI, Vasculitis Damage Index, LDL, low-density lipoprotein; HDL, high-density lipoprotein.

**Table 2:** Occurrence of MACEs according to the number of CVRFs present in patients from the OSTEOVAS cohort.

Number of CVRFs	Number of Patients without MACEs	Number of Patients in Whom ≥1 MACE Occurred, *n* (%)
0	20	1 (4.8%)
1	21	1 (4.5%)
2	28	2 (6.7%)
≥ 3	18	12 (40%)

CVRF, cardiovascular risk factor; MACE, major cardiovascular event. Cardiovascular risk factors included age >50 for men or >60 for women, medical history of cardiovascular disease, smoking status (current or former), obesity (body mass index ≥ 30 kg/m^2^), diabetes mellitus, dyslipidemia, hypertension, or a sedentary lifestyle.

**Table 3:** Impact of each cardiovascular risk factor on the occurrence of major cardiovascular events during follow-up in ANCA-associated vasculitis patients from the OSTEOVAS cohort. The multivariate analysis was adjusted for the use of glucocorticoids and cyclophosphamide.

Cardiovascular Risk Factor (CVRF)	Patients Who Had MACEs among Those with This CVRF, *n* (%)	Patients Who Had MACEs among Those without This CVRF, *n* (%)	*p*-Value	OR (95% CI)
Older age (>50 for men; >60 for women)	14 (30.4%)	2 (3.5%)	0.001	14.71 (2.98–72.68)
Personal medical history of CVD	5 (45.5%)	11 (12%)	0.007	6.54 (1.66–25.71)
Sedentary lifestyle	7 (35%)	9 (10.8%)	0.011	4.50 (1.42–14.29)
Hypertension	13 (24.1%)	3 (6.1%)	0.017	5.04 (1.33–19.12)
Dyslipidemia	6 (31.6%)	10 (11.9%)	0.03	3.86 (1.14–13.09)
Obesity	4 (23.5%)	12 (14%)	0.32	1.93 (0.53–7.00)
Diabetes mellitus	2 (28.6%)	14 (14.6%)	0.34	2.38 (0.40–14.06)
Ever smoker	8 (18.6%)	8 (13.3%)	0.44	1.53 (0.52–4.47)

MACEs, major cardiovascular events; OR, odds ratio; CI, confidence interval; CVD, cardiovascular diseases.

**Table 4:** Factors associated with the occurrence of major cardiovascular events during the follow-up found in the multivariate analysis of the OSTEOVAS cohort.

	OR (95% CI)	*p*
Number of cardiovascular risk factors	1.74 (1.28–2.37)	<0.001
Use of glucocorticoids	1.16 (0.50–2.72)	0.733
Use of cyclophosphamide	0.56 (0.17–1.85)	0.339

OR, odds ratio; CI, confidence interval. Cardiovascular risk factors included age >50 for men or >60 for women, medical history of cardiovascular disease, smoking status (current or former), obesity (body mass index ≥ 30 kg/m^2^), diabetes mellitus, dyslipidemia, hypertension, or a sedentary lifestyle.

## Data Availability

Data could be available on reasonable request.
